# Structural and morphological dataset for rf-sputtered WC-Co thin films using synchrotron radiation methods

**DOI:** 10.1016/j.dib.2019.104383

**Published:** 2019-08-12

**Authors:** R.R. Phiri, O.P. Oladijo, H. Nakajima, A. Rattanachata, E.T. Akinlabi

**Affiliations:** a Department of Chemical, Materials and Metallurgical Engineering, Botswana International University of Science and Technology, Private Bag 16, Palapye, Botswana; b Department of Mechanical Engineering Science, University of Johannesburg, Kingsway Campus, Johannesburg, South Africa; c Synchrotron Light Research Institute, Nakhon Ratchasima, 30000, Thailand

**Keywords:** WC-Co thin films, X-ray photoelectron spectroscopy (XPS), Grazing incidence X-ray absorption spectroscopy (GI-XAS), Synchrotron radiation, SEM

## Abstract

Control and manipulation of synthesis parameters of thin film coatings is of critical concern in determination of material properties and performance. Structural and morphological properties of rf-sputtered WC-Co thin films deposited under varying deposition parameters namely, substrate temperature and rf power are presented in this data article. The surface morphology, crystallite size and nature were acquired using x-ray photoelectron spectroscopy (XPS) and Grazing Incidence X-ray absorption spectroscopy (GI-XAS). Furthermore, Synchrotron findings are correlated with complimentary data acquired from Scanning electron microscopy (SEM), Raman spectroscopy and surface profilometry to predict and point out optimum synthesis parameters for best properties of the film.

**Table:** Specifications table

Subject area	*Materials Science*
More specific subject area	*Thin film technology &Synchrotron radiation characterization*
Type of data	*Table & figure*
How data was acquired	*Synchrotron Light Research Institute, Beamline 3.2Ua (PES) and Beamline 1.1W (XAS)- Thailand,* *Quanta 450 SEM, Senterra Raman spectroscopy & Bruker contour GT profilometer*
Data format	*Raw, analyzed.*
Experimental factors	*10 minutes ultrasonic cleaning with ethanol.* *5 minutes sputter etching with argon ions.*
Experimental features	*Shift in binding energy peaks realized for samples deposited under different rf power values.* *Notable changes in surface morphology observed for variation in substrate temperature,*
Data source location	*Synchrotron Light Research Institute (SLRI), Nakhon Ratchasima, 30000 Thailand*
Data accessibility	*Data is with this article*

**Table:** 

**Value of the data** • The data provides insight on the use of light source methods using incidence at small angles as an alternative for characterization of thin films. • The structural and morphological changes observed for varying deposition parameters may assist in the modelling and parameter selection of rf sputtering of thin films for a wide range of applications. • Synchrotron data can be compared to classical method data provided to further evaluate and validate the crystalline nature of the film.

## 1 Data

Mild steel substrates were coated with WC-Co powder using RF magnetron sputtering using the parameters given in Table 1 Commercial argon (Ar) of 99.9% purity was used as the sputtering gas and kept constant at a gas flow rate of 11 sccm throughout the process. The distance between the target and the substrate was kept at 13cm.

**Table 1:** Data showing sputtering parameters used.

Sample	Time (min)	Substrate temperature (°C)	Rf power (W)
WC-1	120	80	150
WC-2	120	80	200
WC-3	120	80	250
WC-4	120	80	300
WC-5	120	44	200
WC-6	120	70	200
WC-7	120	90	200
WC-8	120	110	200

## 2 Experimental design, materials, and methods

### 2.1 Sample preparation

Samples were magnetron sputtered thin films of WC-Co on 3mm thickness low carbon steel (mild steel) substrate. Eight samples were produced, four for temperature variation (44 °C, 70 °C, 90 °C and 110 °C) and four for rf power variation (150W, 200W, 250W and 300W). Samples were then cut to 10 mm × 5 mm, these dimensions are usually the best for a large variety of equipment holders. Prior to each test the samples were cleaned ultrasonically in ethanol for 10minutes. 5 minutes sputter etching of the samples was performed by argon ions at base pressure of 2.1E-6 Torr and 1KeV power see Table 2, Table 3.

**Table 2:** XPS settings for the wide scan of WC-Co samples.

Grating	1200	lines/mm
PE	650	eV
WF	4	eV
Char	0	eV
Start KE	50	eV
End KE	650	eV
Step KE	1	eV
# scan	1	times
Offset	0	1

**Table 3:** XPS spectra intensities for different deposition parameters with respect to kinetic and binding energy.

Kinetic Energy	Binding Energy	150W	200W	250W	300W	44 °C	70 °C	90 °C	110 °C
		Intensity							
50	596	25.06166	12.1256	64.75534	16.49675	102.5499	35.11037	140.4056	39.25154
51	595	25.68211	11.40452	64.36292	16.53451	100.5216	34.79258	140.2683	38.77942
52	594	25.66903	11.86022	62.56294	16.37758	99.53765	35.58617	138.3303	37.77578
53	593	25.53628	11.58385	62.49327	14.97145	98.38594	34.7693	137.7276	37.21734
54	592	26.5547	11.32083	62.05747	14.68402	98.85297	34.94643	135.7281	36.97716
55	591	25.13718	10.13997	62.12606	14.36097	95.43448	34.58265	133.4917	36.26445
56	590	25.04889	10.69022	59.7716	13.43677	95.5579	33.21706	133.9368	36.27171
57	589	24.43871	10.51585	58.52968	14.79459	95.78153	32.57812	130.3429	34.90149
58	588	23.66046	9.901874	58.26379	13.71759	94.13852	32.75382	126.9245	34.99943
59	587	22.41198	9.86276	54.04884	13.34189	92.51957	31.99853	122.7323	34.73323
60	586	21.36605	10.21684	53.46523	12.88876	87.08629	31.3238	119.5091	34.06188
61	585	21.0701	9.654724	52.23271	13.00851	88.07621	29.77514	118.6031	32.31757
62	584	22.26432	9.250297	48.52856	12.88893	85.42381	30.94055	115.8601	33.0596
63	583	22.03339	9.593023	48.86944	12.21242	84.48134	31.24316	110.5951	32.60296
64	582	21.08886	8.978742	48.37833	12.61727	85.04211	29.61189	109.3529	31.68354
65	581	20.85684	8.874054	45.68193	11.42806	81.77116	28.61278	108.7346	32.17998
66	580	21.28378	9.151168	44.8868	11.7992	80.5078	28.38732	107.1531	31.58782
67	579	20.16332	8.423588	44.30539	11.41385	80.78302	27.26997	103.7635	30.69476
68	578	19.62716	8.546442	42.7994	11.65245	80.11384	27.61681	102.0999	30.71837
69	577	19.55539	8.197529	41.54775	11.49622	77.77612	27.94645	102.1423	30.71145
70	576	19.0947	7.83964	40.66487	10.9883	78.21415	26.95916	99.62709	31.11559
71	575	17.79376	7.888093	41.38212	10.84364	77.64328	27.14986	100.2206	29.12842
72	574	19.38033	7.465156	39.95646	10.841	78.38726	27.89934	99.41694	28.89865
73	573	18.42881	8.352745	38.95651	10.90531	77.96862	26.80715	99.43674	29.75744
74	572	18.71926	7.196319	39.13754	10.72622	76.98383	26.25053	97.14089	29.21969
75	571	19.44963	8.034361	37.85914	10.73792	76.03014	26.52553	97.15982	29.10289
76	570	18.78296	7.913499	38.55026	10.53486	76.5851	26.50143	95.6329	28.72275
77	569	17.85274	7.27137	36.92123	10.34843	74.24982	26.46517	94.05346	28.56035
78	568	18.28865	7.388988	36.45676	10.08604	74.15839	25.64766	96.05712	28.21962
79	567	17.8197	7.438159	36.42453	10.13887	74.72789	24.65872	94.98999	28.29091
80	566	17.50371	7.691382	36.15285	10.18732	74.50383	24.74632	92.80082	26.10704
81	565	17.81004	7.718651	35.07711	9.843765	73.79402	25.10948	92.38518	26.98157
82	564	17.67604	7.587327	36.32271	10.12373	71.67751	24.57625	91.83105	27.50313
83	563	17.43912	7.226074	34.16521	9.435246	72.27693	24.58131	89.50601	26.80952
84	562	17.5737	7.104797	33.11676	9.222501	71.43933	25.13187	89.89986	27.42294
85	561	17.4017	7.027733	33.46149	9.613071	71.44082	24.22903	88.26237	26.33146
86	560	17.27645	7.445585	31.97046	9.722328	71.3241	24.33255	88.37644	25.31324
87	559	17.06095	7.452146	33.1236	9.277317	68.47763	24.09194	87.5796	25.75849
88	558	17.07174	7.262771	33.06543	9.647137	69.75322	23.99329	86.72899	27.16974
89	557	16.64531	6.707751	31.37053	8.713446	70.85045	23.37598	87.70277	26.32821
90	556	16.6498	6.635196	31.05939	9.34779	71.30265	23.12893	85.36696	25.10152
91	555	16.62292	6.796544	32.42901	9.117887	69.50985	24.043	85.97819	25.81155
92	554	15.85067	6.893685	31.08327	8.395345	69.38568	23.21276	86.63255	26.06232
93	553	16.33966	6.521663	30.40704	8.981106	69.06602	22.9832	83.62133	24.02072
94	552	15.86225	6.64382	30.70043	8.393933	68.96664	23.31322	85.80173	25.4812
95	551	16.36525	6.729616	31.34207	9.344351	70.46588	22.07609	83.21405	24.87108
96	550	15.90143	6.558332	30.51886	8.701416	68.24706	22.89702	82.90493	24.67719
97	549	15.63134	6.612746	29.37676	8.535627	69.85049	22.76854	84.23777	25.11108
98	548	15.91656	6.6838	28.68052	8.033379	69.33187	22.10805	82.71617	24.589
99	547	15.85445	6.767866	30.00218	7.898322	68.78852	23.22671	81.58383	24.97885
100	546	15.94253	6.250193	28.2639	8.951084	67.85097	22.40184	82.60806	23.68439
101	545	16.10112	6.339115	29.50433	8.186521	68.41497	22.1971	81.84357	24.80344
102	544	16.01631	6.508946	28.80966	8.533837	67.35213	21.97746	81.11088	23.92011
103	543	15.80809	6.128909	27.99398	8.376741	66.66298	22.0035	81.64768	23.53644
104	542	15.29076	6.165849	27.79082	8.124467	66.28574	23.43635	80.47849	23.68502
105	541	14.85522	6.269184	26.7021	8.025619	67.11051	22.13109	79.4267	24.0949
106	540	14.60891	6.647758	27.69596	7.949139	67.81352	21.60717	77.61318	24.61717
107	539	15.69819	5.518671	26.61289	7.879126	67.26297	21.91638	78.01245	23.45207
108	538	14.38345	6.073772	26.30781	7.145234	65.47841	22.16856	78.19402	22.2538
109	537	13.91606	6.395863	25.72895	7.503797	66.37522	22.76881	77.39411	22.99122
110	536	16.02159	5.846221	25.37794	7.965605	66.62892	22.3064	75.50236	23.57518
111	535	14.72562	5.945294	25.53072	7.780111	66.20706	21.18824	78.01152	23.75874
112	534	14.70245	6.361792	25.65491	7.529166	65.56734	22.10626	80.01716	23.61903
113	533	15.45565	6.080509	26.4354	8.454758	66.36747	23.05921	80.04787	25.1909
114	532	16.08464	6.487797	27.7401	8.122526	73.44674	22.62169	84.22404	26.74919
115	531	16.06564	7.011224	28.9815	9.074915	75.22357	23.66814	83.60382	26.39546
116	530	15.97414	6.641095	31.14606	8.123885	73.06541	22.49508	86.74489	23.67965
117	529	15.56016	5.585814	28.92408	7.883232	68.88378	21.54682	84.76082	22.53195
118	528	14.81964	5.663847	25.31871	7.739561	65.85766	21.46096	76.27967	22.29653
119	527	14.66105	5.581334	24.46194	7.399497	63.97488	20.93395	73.49493	21.37305
120	526	13.45276	5.894289	22.72052	7.390769	64.01524	20.16011	72.69125	21.66264
121	525	13.37252	5.711878	23.1897	6.681268	63.3808	20.61111	73.37031	22.21702
122	524	13.50539	5.7874	23.36432	6.975499	63.88053	20.7144	73.94929	21.77001
123	523	13.16612	5.676921	22.90021	7.527079	64.64924	20.5782	73.15376	22.17061
124	522	13.21485	5.891895	22.85105	7.544885	62.15591	19.77938	73.56075	23.09759
125	521	13.6858	4.994095	21.83828	6.897217	64.1474	20.85461	72.66213	21.24263
126	520	13.81201	5.431336	22.9312	7.352662	64.41463	21.17746	72.70888	22.44371
127	519	13.2602	5.501437	21.15224	6.952043	64.67835	20.01183	73.0953	22.44192
128	518	13.761	5.865968	22.27366	7.082473	64.60144	19.58162	71.76568	21.4665
129	517	13.10868	6.006255	21.60996	6.95922	64.37073	20.13942	74.31489	21.86729
130	516	13.5698	5.587309	22.99909	6.581441	63.30525	20.30249	72.20908	21.60958
131	515	13.56831	5.617649	21.88953	7.420295	64.60837	19.9669	72.41401	21.38215
132	514	12.67972	5.321791	21.93176	6.762587	64.14324	20.37259	72.61843	21.21665
133	513	13.01142	5.479942	21.21076	7.51942	62.67134	20.91094	71.00963	21.15573
134	512	12.80656	5.46731	22.00456	7.226709	63.52302	20.47872	71.86075	22.15474
135	511	13.37025	5.652291	20.9772	7.400403	62.27312	19.82767	71.41906	20.9625
136	510	12.93516	5.271795	21.53711	6.830202	63.74945	19.65905	70.15544	22.25174
137	509	13.37494	5.755574	20.60929	7.175847	63.34452	19.35581	71.09517	21.63426
138	508	12.68561	5.48875	21.08721	6.88595	64.59621	19.30528	71.84477	21.60959
139	507	12.69146	6.169845	21.13008	7.118192	64.09763	20.59597	70.20263	21.35086
140	506	12.50781	4.955345	20.61494	6.90317	63.92092	19.852	69.82386	20.69669
141	505	12.9221	5.492251	20.15835	6.347999	62.9682	19.84398	70.67922	20.96154
142	504	12.95821	5.507738	20.78878	6.6956	64.12724	19.78613	72.08068	21.31885
143	503	13.14204	5.124575	20.08544	6.844269	64.05771	19.95075	70.70665	20.3546
144	502	12.6644	5.325233	20.20034	6.6698	64.25444	19.80249	71.12656	20.88049
145	501	13.02773	5.559324	20.55918	6.940964	64.35166	19.64023	71.54489	21.13598
146	500	13.64839	6.099368	19.51939	6.78026	64.58344	19.17519	71.01376	20.81357
147	499	12.69313	5.269316	19.40847	6.818426	63.68433	19.42329	70.25844	20.87561
148	498	13.24994	5.076658	20.1568	6.52617	63.29366	19.01095	70.71461	21.40021
149	497	12.08417	5.560761	19.09407	6.873308	63.77805	18.98174	71.43637	20.44668
150	496	12.11028	4.927951	20.39725	6.166531	64.48302	19.06264	70.24118	21.0074
151	495	11.8978	5.505875	19.8212	6.757664	64.33043	18.77706	69.28268	21.22713
152	494	12.3587	4.870759	18.88041	6.398765	63.73684	19.69453	68.04326	21.19405
153	493	12.58202	5.28865	19.84991	6.958021	64.14796	19.17694	70.24084	20.90814
154	492	13.16525	5.276512	19.173	6.830428	62.90794	19.11515	70.00034	20.1576
155	491	12.56212	5.354019	18.91595	6.327805	63.04383	19.23995	71.52617	20.99277
156	490	12.52117	5.325018	18.70862	6.093044	63.46818	18.85566	70.21663	20.64243
157	489	12.54194	5.066276	19.08266	6.594387	63.97733	18.72992	70.53307	21.25433
158	488	12.38564	5.014129	17.97955	6.473818	62.22866	19.0824	68.90622	20.26883
159	487	12.32423	5.381996	18.6375	6.589	63.87122	18.66979	69.11837	20.80175
160	486	11.92774	5.202336	19.00052	5.657967	65.0977	19.1226	69.75397	20.50274
161	485	11.65393	4.887589	19.04464	7.087881	64.44875	18.77838	69.10759	20.99006
162	484	11.66011	5.297093	18.76965	6.488485	65.71615	19.32957	67.95137	19.26593
163	483	12.12968	5.205538	17.99632	6.561574	65.56562	19.05372	69.492	19.61914
164	482	12.32997	4.94096	17.99585	6.086966	64.43314	18.91474	66.976	19.66454
165	481	12.41892	5.07668	18.64635	6.228212	65.07647	19.4348	66.85045	20.31304
166	480	12.1409	5.129359	18.37095	5.851748	65.34857	18.45863	69.81057	20.30003
167	479	11.81288	5.665365	17.81252	6.357145	64.18246	18.91529	69.93981	20.09463
168	478	11.77529	5.272307	18.06988	6.02611	64.12123	18.64086	68.57672	20.25467
169	477	11.76048	4.960765	17.85003	6.518417	64.96618	18.18961	68.11993	20.39079
170	476	11.65436	5.340714	17.30046	6.654423	63.85397	18.07201	68.26479	19.84618
171	475	11.43997	5.139066	18.24472	6.163513	64.70979	18.63801	67.63825	20.25699
172	474	11.48928	5.082631	17.78972	5.988578	64.38247	18.42575	68.25688	19.92708
173	473	12.19005	5.434717	17.49832	6.209181	64.09872	18.02846	68.23844	19.84247
174	472	11.3018	4.90267	17.14509	5.928772	64.19811	17.93788	70.78495	19.54258
175	471	11.93451	5.309129	16.95157	6.524763	64.57519	19.30929	70.31233	19.60295
176	470	12.21511	5.36414	16.64	6.375384	64.92451	18.46131	70.40554	20.47236
177	469	12.02138	5.124799	18.69056	6.650038	64.43054	18.24304	70.77166	19.8385
178	468	11.41981	5.53725	17.71081	5.615993	64.97667	18.85372	70.03955	19.98137
179	467	11.54624	4.90068	17.76729	6.174046	63.8736	18.73187	68.44356	19.94374
180	466	11.65413	5.378793	16.74821	6.259742	65.13962	18.47305	70.55533	20.3795
181	465	12.29871	5.126238	17.31329	6.45378	64.31664	18.50169	69.11888	19.81456
182	464	11.44858	4.889172	17.55648	6.199576	64.29118	18.48979	69.1105	20.52317
183	463	11.40102	4.910835	16.8256	5.762983	65.36185	18.16523	68.98076	19.50708
184	462	11.8652	4.992474	17.3516	6.202132	64.61908	18.40969	69.39347	20.69301
185	461	11.87601	5.119181	17.37182	6.244749	62.31757	18.18011	69.45385	19.80448
186	460	11.54151	5.499876	17.19457	6.252373	64.89005	18.13803	70.41044	19.88628
187	459	11.46791	5.328464	17.08193	6.210312	63.5457	18.9049	68.59103	19.60353
188	458	10.71689	4.868583	16.46482	6.079936	64.90903	18.92493	67.94832	20.30337
189	457	11.56291	5.538557	16.57986	5.890181	64.43576	18.28269	67.68841	20.121
190	456	12.29925	5.162683	16.38393	5.945487	65.11932	18.27512	68.58484	19.3641
191	455	11.67737	4.968081	16.36834	5.876898	64.81195	18.53659	68.35217	20.61699
192	454	12.56343	4.974471	16.15555	5.802143	64.2934	18.51067	69.22564	20.67279
193	453	12.62966	4.483975	16.89339	5.773846	63.72987	18.13027	69.13127	19.95059
194	452	11.44958	4.767855	16.10285	5.95777	64.4016	18.6081	69.30956	21.2383
195	451	11.51114	5.412323	16.19366	5.864062	65.71216	18.30996	70.54825	20.07736
196	450	12.12352	4.96499	16.82057	5.927906	63.72131	18.40976	68.10943	21.01339
197	449	11.54364	5.072962	16.39132	5.870499	65.20698	18.44702	70.30729	20.48828
198	448	11.93786	5.137744	16.72003	5.841153	64.15467	18.164	69.20147	20.30269
199	447	10.70172	5.24935	16.27451	5.800435	65.01922	18.70704	70.11161	19.70215
200	446	11.45538	5.103818	15.91107	6.043179	65.14708	19.23928	68.88122	20.21938
201	445	12.04374	5.199619	16.98676	5.912249	66.0585	18.72155	67.99772	20.75863
202	444	11.49532	4.949361	16.83135	6.470824	64.91385	19.27043	69.35403	20.09393
203	443	12.16439	5.452686	16.38792	6.069382	66.38892	18.61546	70.50399	19.9605
204	442	11.8808	5.380904	16.40292	6.168275	66.16438	18.14085	70.00631	20.03136
205	441	11.54466	5.293806	16.97325	5.861595	64.68741	18.37682	69.93295	20.28531
206	440	11.53122	5.444568	17.00099	5.930168	65.47536	19.28541	71.2956	20.23879
207	439	11.86809	5.027975	16.36428	6.027631	65.81195	18.40737	70.90321	20.08246
208	438	12.10818	5.135063	16.58947	6.02459	64.36151	19.69874	70.09822	20.29387
209	437	11.17111	5.073582	16.61965	6.093507	64.35424	18.30599	69.54264	20.07885
210	436	11.77276	5.394737	17.33693	6.012248	65.51653	19.19649	71.11027	20.09103
211	435	11.94031	5.099392	16.63055	6.390153	66.2774	18.92518	70.92755	20.58397
212	434	11.48298	5.179607	17.50423	6.545379	65.46331	17.97506	71.10531	20.33811
213	433	12.04934	4.929892	17.02865	6.606672	66.92892	19.91938	71.26443	21.06481
214	432	11.13192	5.122936	16.97763	5.874405	67.91913	19.30862	70.62672	19.74241
215	431	11.50893	5.11585	17.53604	6.553069	66.14283	18.57037	71.00953	20.64762
216	430	11.62459	5.508563	16.05609	6.074658	66.69569	18.57634	71.28579	21.0035
217	429	11.9515	5.260893	16.94245	6.689945	66.04403	18.84792	70.82458	19.72105
218	428	11.69849	5.38215	16.72957	5.460359	65.97384	19.02767	72.15795	20.79276
219	427	12.23911	5.077093	17.79859	6.338617	65.12026	18.59249	70.74409	20.7176
220	426	11.75068	5.422701	16.44742	6.274731	65.96288	19.05795	71.21548	20.18315
221	425	11.16984	5.285593	17.16495	6.262877	67.60053	18.71904	72.7106	21.0929
222	424	12.35081	4.800278	17.41011	6.053988	67.83376	19.80597	70.90754	20.13747
223	423	11.80323	5.50226	16.83147	5.727689	67.8876	18.27847	69.86458	21.05416
224	422	11.79282	5.477549	15.92663	6.608007	67.04081	18.48126	71.56525	20.13959
225	421	11.8947	5.447163	17.00769	5.92155	68.63957	18.9917	71.84776	20.02648
226	420	11.52287	5.09042	17.07319	5.554725	68.15118	19.48341	72.75407	20.97508
227	419	11.52114	5.040864	17.31149	6.346206	69.63459	19.54994	72.52513	20.46032
228	418	12.33556	5.591237	17.13103	6.45378	68.65576	18.9613	73.01002	21.4622
229	417	11.49555	5.407032	16.51416	5.915825	68.35771	18.8634	71.87512	20.53576
230	416	12.60244	5.527195	16.70782	5.612014	69.29464	18.90676	71.88102	21.2413
231	415	12.16212	5.717603	16.48387	5.844098	68.17418	19.11729	71.6285	20.43158
232	414	12.22683	5.54772	17.0552	6.362358	69.97751	19.57163	75.36028	21.64884
233	413	11.99387	5.723828	16.58342	6.24134	69.6526	19.93185	74.10856	21.08232
234	412	12.00575	5.432114	17.3714	6.834567	69.99148	19.06766	73.59888	22.04093
235	411	12.25736	5.649169	17.93279	6.491581	69.8254	19.96046	74.68986	20.97229
236	410	11.26147	5.8412	17.80558	6.311418	70.58965	19.1893	75.18135	21.92986
237	409	12.59542	5.954366	17.48408	6.782529	71.16498	19.49754	76.1103	21.30091
238	408	12.23965	5.694778	16.54363	6.188734	72.14325	19.89307	76.62021	20.95453
239	407	13.33905	5.931925	16.6787	6.802698	72.15019	19.41008	75.78174	21.48846
240	406	12.57968	5.942542	17.23625	6.362753	70.45513	19.8397	75.81274	20.76316
241	405	12.99021	5.364922	16.99058	7.032738	71.17286	20.01864	74.70287	22.12215
242	404	13.00215	6.027105	17.9516	6.450862	70.08217	20.21549	76.12811	22.43143
243	403	12.67031	5.464088	17.84571	6.696714	71.37448	19.51879	77.95716	21.54416
244	402	11.65268	6.101138	17.88559	7.166885	70.77146	20.65678	77.19474	21.08633
245	401	12.56815	6.04843	17.27956	6.351995	74.12501	20.02449	75.76205	20.97653
246	400	13.0773	6.107832	18.14837	6.555286	71.65591	20.21192	79.01344	20.92769
247	399	13.15373	5.64527	18.08619	6.823564	71.88585	20.20728	77.66665	21.84579
248	398	13.26033	6.195484	17.9021	6.612902	74.6352	20.54687	78.66925	22.64544
249	397	13.1355	6.156335	17.38447	6.673562	75.2527	20.38372	78.78826	23.13769
250	396	12.60134	6.523002	18.8161	6.713323	74.56164	21.05231	78.89529	23.32454
251	395	12.93021	5.881225	18.0671	6.918226	75.03274	20.7208	80.47767	22.51358
252	394	13.32134	5.694581	18.57372	6.971004	76.42185	20.87201	80.24373	22.72574
253	393	12.91588	6.082664	19.13197	6.84297	76.31739	21.69918	82.28265	23.08947
254	392	14.133	6.150525	18.90287	6.796272	74.71113	21.05515	81.19149	22.52361
255	391	14.21227	6.353889	18.38781	7.70532	75.22656	22.18559	81.16744	22.34183
256	390	13.38715	6.200495	17.83857	6.9104	77.04075	21.27794	79.86536	22.12983
257	389	13.04525	6.283538	18.53477	6.753785	75.5597	21.41305	79.6332	22.02612
258	388	13.07809	5.876496	18.37332	6.804525	73.88479	20.31313	80.86624	21.27532
259	387	12.95764	5.76915	18.81162	7.110772	74.87244	20.34794	79.50173	21.79033
260	386	13.33089	5.530095	18.13421	6.677456	71.63681	20.83506	76.91123	21.35251
261	385	12.83447	5.527437	18.44287	6.371857	69.65532	19.03246	77.32332	20.9834
262	384	12.58153	5.157318	17.51653	6.345766	67.49663	19.15175	75.36328	19.31475
263	383	11.54248	5.427483	17.83996	6.351827	65.37393	18.81684	74.07182	19.87942
264	382	11.9808	4.798978	17.26246	5.627604	63.60894	17.58499	71.07487	18.17771
265	381	11.10292	4.56873	16.67318	5.984795	60.28231	16.75276	68.16399	18.03324
266	380	10.38435	4.754024	15.39843	5.487743	55.89961	15.86667	64.37028	15.96539
267	379	11.20869	4.120434	15.67759	5.303014	53.19449	15.49219	61.85561	14.85557
268	378	9.890161	3.585741	15.16646	5.746568	50.73761	13.70006	59.3207	14.81447
269	377	9.796656	3.61027	14.60577	4.944219	49.53238	13.85216	55.97199	12.83924
270	376	8.353547	3.308642	14.64657	4.709591	46.72131	12.54752	55.59867	13.17334
271	375	8.70045	3.346118	13.60815	4.764334	43.97251	12.09811	50.51412	12.33564
272	374	9.246976	3.18206	13.64544	4.548539	43.27801	11.14809	49.75755	12.10096
273	373	8.663807	3.344913	13.09863	4.424364	41.72223	11.59545	48.18135	11.88553
274	372	7.86547	3.284717	13.7194	4.085354	40.37327	10.61157	47.71629	11.79499
275	371	8.107925	3.07604	12.57637	4.560937	40.03565	10.57751	45.08189	11.23247
276	370	7.923839	3.390161	13.26108	4.08501	40.33269	10.48999	46.5238	11.07598
277	369	7.315684	2.91363	12.07403	4.358445	37.71473	10.04788	44.50195	10.44814
278	368	7.902785	3.232981	11.37202	4.562089	38.62706	9.966525	44.09272	10.6675
279	367	6.992477	2.906033	12.63528	4.009513	38.51408	9.672645	44.20194	10.49387
280	366	7.498106	3.136913	11.9579	4.048036	38.16363	9.932035	44.46406	10.15327
281	365	7.308317	3.391265	11.8539	4.249844	37.48823	10.19453	42.74547	10.69796
282	364	7.603762	2.628151	11.65811	4.073839	36.96705	9.98472	42.84108	10.5198
283	363	7.435188	2.570913	11.9856	4.47766	37.50639	9.787005	43.9682	10.2236
284	362	7.230005	3.104082	11.54505	4.320523	37.54274	9.953945	43.5977	9.971868
285	361	7.153836	3.05066	11.3298	4.158678	36.84967	9.733206	42.56884	10.10279
286	360	7.104522	2.887801	11.41162	4.015656	37.30643	9.559422	42.89197	9.266908
287	359	7.282919	2.964581	12.41664	3.933987	37.24653	9.883226	43.20673	9.945932
288	358	7.334451	3.333727	12.50662	3.972955	37.03306	9.974502	43.65339	9.777319
289	357	6.964024	2.817957	12.36251	3.782405	36.73307	9.713965	42.53731	9.916923
290	356	7.215729	3.177462	12.04289	4.178329	37.88631	9.905082	43.03367	10.01876
291	355	7.027303	2.775365	12.27041	4.346462	36.08676	9.665981	42.97943	10.04339
292	354	7.140648	2.914936	12.39865	3.845092	36.12049	9.34369	42.29024	9.706725
293	353	7.093623	3.462544	11.36415	4.267997	34.65205	9.113914	41.55368	9.869122
294	352	6.632094	2.654015	11.57591	3.815442	35.35259	10.07255	41.62616	9.903694
295	351	7.403015	2.69755	11.51298	4.573256	35.17527	9.439419	41.62469	9.341695
296	350	7.216674	2.58013	11.17452	4.172458	36.43185	9.764969	41.39541	9.718076
297	349	6.888386	3.047183	11.83448	4.294611	35.4993	10.04238	42.39544	10.2225
298	348	7.308473	2.949846	11.6382	3.961596	35.58083	9.738424	42.09859	10.00313
299	347	7.037491	2.942327	11.31529	3.866708	36.06691	9.502154	41.54437	9.301717
300	346	7.751887	2.661702	11.20484	4.028031	37.16083	9.758028	41.23419	10.37151
301	345	6.706204	2.912247	11.18946	4.244886	35.97519	9.750131	41.5851	9.145505
302	344	7.344761	2.939807	11.45829	3.864916	34.35199	9.670929	41.0367	9.310621
303	343	6.953348	3.063487	11.35159	4.437429	35.33219	9.638901	41.72235	9.731954
304	342	7.018988	3.060383	11.47712	3.993639	34.9861	9.496858	40.76669	9.533377
305	341	6.515395	2.922123	11.95598	4.358994	34.03005	9.815667	40.46869	10.00805
306	340	6.698554	2.839019	11.59867	3.827254	34.51312	9.354942	40.29808	9.966647
307	339	6.597359	2.832453	11.5907	4.265311	34.6923	9.232626	39.98102	9.908833
308	338	6.716778	2.693595	11.8702	4.043497	33.97694	9.247462	40.90962	9.287295
309	337	7.155201	3.020216	11.92552	4.356794	33.80244	9.262011	40.18115	9.347766
310	336	7.063114	2.612935	11.65078	4.181914	34.02085	9.356931	40.41464	9.316454
311	335	6.985744	2.570913	11.0022	4.195974	33.97567	8.7253	39.25359	9.02457
312	334	7.191959	3.06386	11.95095	3.803395	34.67517	9.381862	40.49809	9.528448
313	333	6.867044	3.036309	10.69215	3.880292	33.93289	8.982019	39.83544	9.487582
314	332	7.283143	2.434391	10.98865	4.033576	33.87106	8.942899	38.87772	9.30471
315	331	7.341433	2.828491	11.87245	4.146676	33.96266	9.273273	38.85504	9.065928
316	330	6.58724	2.896257	11.2147	4.178554	34.47856	8.857336	39.96565	8.561443
317	329	6.931542	3.08823	11.32384	4.091759	33.54785	9.306065	37.51229	8.830156
318	328	6.999838	3.014534	12.09362	4.468421	32.93238	8.990789	38.81597	9.40324
319	327	6.921414	2.568306	11.93988	4.074355	32.48769	9.103384	38.16726	9.178205
320	326	6.770791	2.404693	10.42456	4.159904	34.30757	8.631486	39.40727	9.617687
321	325	7.11014	2.56814	11.26087	4.0187	33.13883	8.667707	38.74474	9.026924
322	324	6.075266	2.998188	11.9104	4.19686	32.31044	9.107451	38.34005	8.584503
323	323	6.545642	2.860867	11.67854	3.903952	31.44204	8.723732	37.43054	8.912322
324	322	6.324444	2.87161	11.38694	3.785116	33.07562	8.543813	37.26214	8.973867
325	321	7.019542	2.719676	11.97815	3.798102	32.81522	8.181077	37.09154	8.89544
326	320	6.658548	3.079169	10.9953	4.122266	31.9748	8.913152	39.03034	8.885321
327	319	7.006959	2.893282	11.79465	3.535939	32.18055	9.152403	38.6852	8.703859
328	318	7.154887	2.531785	10.96762	3.911852	32.26876	8.717785	38.87657	8.949091
329	317	6.587896	2.912087	11.27436	3.829511	32.75107	8.537686	37.87388	9.122105
330	316	7.048953	2.732432	11.60348	4.090376	31.67972	8.944406	36.54997	8.91917
331	315	7.343577	2.750794	11.73347	4.35289	31.89729	8.98742	38.24067	8.357762
332	314	6.982899	3.089345	11.33722	3.858098	32.07843	9.007277	39.05743	8.980902
333	313	6.340359	2.874423	11.45544	4.230026	31.90286	8.968515	38.70356	9.07498
334	312	6.950152	2.586224	11.38357	4.041729	31.85547	8.593992	38.20404	8.629585
335	311	6.860729	2.633509	10.87342	3.888955	32.09906	8.20354	37.31079	9.041682
336	310	6.923745	3.142153	10.933	3.778267	31.9187	8.585252	37.87057	8.872308
337	309	6.915847	2.732212	10.87854	3.837326	31.55111	8.860736	36.88892	8.438415
338	308	7.059089	2.495589	11.42825	3.950901	32.24501	9.181345	38.15993	8.584003
339	307	6.784021	2.483197	11.25339	3.880367	30.82713	8.431768	37.9643	8.394604
340	306	6.56382	2.490523	11.04259	3.840235	30.8937	8.393221	37.20084	8.764334
341	305	6.344494	2.868681	11.53098	4.205565	30.04858	8.399654	36.93463	8.211945
342	304	6.313874	2.963257	10.65583	3.548253	30.24006	8.341214	35.55152	8.341945
343	303	5.756008	2.475815	10.90026	4.162182	29.63326	8.199143	35.02903	8.046775
344	302	6.412098	2.741453	10.07145	3.762178	29.97818	8.064687	35.43277	8.166175
345	301	6.426652	2.37114	11.29126	3.434216	28.70015	8.366929	34.50027	8.134001
346	300	6.937023	2.631144	10.25561	3.79937	28.19754	7.721904	34.32405	7.372204
347	299	6.567708	2.346583	10.27688	3.638786	28.11832	6.804068	33.2939	7.123271
348	298	6.335132	2.581497	10.78188	3.762971	26.49914	7.450666	33.16278	7.411735
349	297	6.291184	2.313871	9.967956	4.018863	26.1975	6.976084	32.90672	6.86745
350	296	6.259928	2.149893	10.92141	3.34042	26.60495	6.526706	32.61131	6.389384
351	295	6.149145	2.310735	11.15168	3.635088	26.44322	6.784974	32.31816	6.622941
352	294	5.809445	2.171999	10.48089	3.625417	26.22444	6.85633	32.62791	6.117933
353	293	5.923191	2.160444	10.87809	3.75998	26.40733	6.418621	32.60828	6.4482
354	292	6.276372	2.169144	10.32604	3.684445	26.20794	6.894404	31.61623	5.728883
355	291	5.837323	2.022041	10.15445	3.712386	25.4193	6.077564	32.34072	6.313984
356	290	5.572781	1.91277	10.73922	3.540789	25.03943	6.104184	32.16238	6.232918
357	289	5.960414	2.084321	9.889787	3.359004	25.21913	6.934065	31.69938	6.505585
358	288	6.420212	2.317611	11.45802	3.807209	25.85851	6.581062	31.9712	7.006466
359	287	5.792993	2.408808	10.71886	3.555349	27.69351	6.720126	34.60566	7.168772
360	286	7.732489	3.66195	11.67032	4.216898	33.03534	8.926841	38.04729	10.18488
361	285	10.47821	5.171727	14.66659	6.540684	48.66802	15.02125	56.79259	16.15004
362	284	12.53057	5.561248	17.06333	6.85568	63.51983	21.94652	69.82068	21.19671
363	283	9.049886	3.127372	13.72678	4.870588	52.07148	14.67995	54.81907	14.81341
364	282	6.645903	2.159625	10.6175	3.352867	29.20167	8.16554	31.90807	7.29293
365	281	4.928862	1.853248	10.17043	3.076554	21.37242	5.225686	27.68071	5.163478
366	280	5.114358	1.522297	9.305055	3.323945	19.90127	5.232591	26.76904	5.00677
367	279	5.200604	1.684166	9.516786	3.313007	18.63397	5.152748	27.22234	4.275417
368	278	4.784103	1.762242	9.739901	3.083711	19.03361	5.129427	26.72212	4.516498
369	277	5.292924	1.417574	9.534017	3.079153	20.08196	4.80208	27.08989	4.183641
370	276	4.583923	1.439092	8.825721	3.107076	20.05597	4.95602	25.84772	4.408197
371	275	4.939454	1.741629	9.038776	3.313426	20.56799	4.623009	26.6785	4.464513
372	274	5.060724	2.02706	9.375485	3.327631	20.12224	5.089391	25.98597	4.517363
373	273	4.569599	1.562729	9.579694	3.10891	19.24825	4.888856	26.16789	4.636651
374	272	4.955808	1.668938	10.05059	3.337178	19.7974	5.050859	26.79443	4.297252
375	271	4.335911	1.990597	9.190971	3.134253	19.44879	5.0198	25.80673	4.253928
376	270	4.757742	1.715539	9.713692	3.111604	19.6749	4.98331	26.61762	4.342785
377	269	4.739429	1.744585	8.68036	3.163175	19.3627	5.045903	25.92558	4.059007
378	268	4.688981	1.672718	9.800807	3.131733	19.66136	4.969306	24.80548	4.615506
379	267	4.778086	1.706811	9.631778	3.351034	18.9213	4.524081	25.83557	4.418744
380	266	4.78264	1.775398	8.59323	3.20318	19.45318	4.855004	26.00465	4.263249
381	265	4.750944	1.787725	9.028289	3.222815	18.91068	4.412348	26.10657	4.411235
382	264	4.596626	1.64853	9.337369	3.126443	19.53438	4.658431	25.42538	4.316298
383	263	4.837273	1.744869	9.789724	2.959642	18.86571	4.849857	25.83382	4.307269
384	262	4.574617	1.769632	9.745831	3.018944	18.06047	4.628038	26.36147	4.396175
385	261	4.9996	1.764949	9.312394	3.263306	19.34394	4.667944	25.84371	4.215729
386	260	4.758161	1.739336	9.25288	3.18185	19.0352	4.532315	25.16896	3.910486
387	259	4.934025	1.510269	9.634042	3.027376	19.30276	4.677615	25.36282	4.506571
388	258	4.304355	1.800357	9.399255	3.145171	18.10796	4.422835	25.24382	3.930489
389	257	5.160686	1.591548	8.975442	3.206782	17.84125	4.652718	25.26752	4.024081
390	256	4.924049	1.761089	9.280291	2.914997	17.96941	4.542545	25.27981	4.101547
391	255	4.717527	1.776916	9.264502	2.908045	18.30829	4.524547	24.78736	4.864825
392	254	4.227098	1.462238	8.832142	2.929054	18.42594	4.800602	25.08564	4.822166
393	253	4.746962	1.871033	9.301868	2.778263	17.68276	4.343247	24.56789	3.931695
394	252	4.557649	1.612971	8.971592	2.936365	18.16292	4.369624	24.81895	4.105075
395	251	4.800962	1.551808	9.132268	2.943825	17.46337	4.861664	24.79097	4.007228
396	250	4.896611	1.575377	9.183618	3.023413	18.0421	4.619381	25.10736	4.041082
397	249	4.478039	1.634274	9.147674	3.012656	18.31544	4.838228	25.27716	4.210869
398	248	4.741255	1.540565	9.526665	2.969422	18.76317	4.37521	25.65066	4.443706
399	247	4.341803	1.806893	9.765577	3.290368	17.41525	4.584251	24.47094	4.263252
400	246	4.657482	1.70702	9.291838	3.260512	18.12936	4.912592	24.62242	4.361627
401	245	4.699701	1.48868	9.500773	3.230856	18.25581	4.302042	24.43211	3.988715
402	244	4.745352	1.556375	9.207506	2.880783	18.00572	4.635832	25.44383	4.213247
403	243	4.482164	1.742169	8.648379	3.158371	18.2206	4.438727	24.42184	3.913137
404	242	4.819819	1.505644	8.570178	2.962021	18.35212	4.286178	24.43307	3.908537
405	241	5.251352	1.61787	8.402064	2.480476	17.73765	4.317835	25.4617	4.224784
406	240	4.521338	1.728749	8.476547	2.813207	18.32221	4.442121	24.81962	3.96585
407	239	4.916594	1.735939	9.186398	2.814159	18.04549	4.442825	24.37325	3.952537
408	238	4.477504	1.365826	8.764002	2.613586	17.79779	4.356276	24.85377	4.408088
409	237	4.60052	1.656547	8.423133	2.603278	17.81013	4.387974	24.04047	4.267212
410	236	4.056625	1.627155	8.402238	2.685147	17.45411	4.37476	48.7857	4.135942
411	235	4.39378	1.705215	8.587174	2.674233	17.54884	4.301453	25.30258	3.70322
412	234	3.918302	1.44352	8.815092	2.909056	17.49182	4.278904	24.24995	4.143638
413	233	4.544525	1.560562	8.239434	2.931408	16.92691	4.250897	23.70855	3.651327
414	232	4.512477	1.444932	8.37904	2.869753	17.71515	4.142462	23.92153	4.119737
415	231	4.20241	1.550225	8.105209	2.67856	18.36659	4.502983	24.38259	4.227018
416	230	5.222102	1.778727	8.097656	2.390446	17.7534	4.224676	24.74256	4.187532
417	229	4.237178	1.688299	8.619564	2.569136	17.70759	4.195792	24.61847	4.132651
418	228	4.436776	1.447704	8.424386	2.51062	17.22213	4.171565	24.13548	3.751796
419	227	4.348108	1.912836	8.39788	2.57865	17.23784	4.483008	23.97595	4.076562
420	226	4.096439	1.643968	8.713018	2.860928	16.78944	4.455151	24.73701	3.990266
421	225	4.606832	1.669962	8.316466	2.515606	17.51827	4.249005	23.62153	3.813669
422	224	4.516965	1.813228	8.455375	2.832766	17.87245	4.400154	25.00149	3.65473
423	223	4.324855	1.410701	8.333179	2.60174	16.63382	4.644355	23.49346	4.264313
424	222	4.469232	1.736577	8.319573	2.642012	17.52162	4.194514	23.17401	3.887915
425	221	4.015952	1.577568	8.944807	2.651274	18.3426	4.294974	23.46619	3.671632
426	220	4.169634	1.366383	9.022009	2.52662	16.94169	4.534627	23.92891	3.707207
427	219	4.673165	1.528693	9.025906	2.473669	17.00338	4.143021	24.78619	3.809588
428	218	3.897555	1.540124	9.263881	2.524386	17.65494	4.151376	23.5032	3.884357
429	217	5.111876	1.586616	8.425708	2.76736	17.22588	4.205872	23.74967	3.920743
430	216	3.91557	1.62197	9.025906	2.485246	17.15563	4.197385	23.32787	3.942848
431	215	3.807056	1.565349	8.735793	2.726064	17.12881	4.342087	23.88799	4.301028
432	214	4.553928	1.595962	8.728813	2.963521	16.66598	4.089248	23.95804	3.996275
433	213	4.309177	1.506505	8.604039	2.908932	17.28091	4.118346	23.61524	3.783726
434	212	4.625292	1.59084	7.924434	2.694567	16.37672	4.030714	23.14682	4.035339
435	211	4.641607	1.209382	7.710842	2.597534	16.98666	4.304899	23.23499	3.81801
436	210	4.103061	1.712317	9.119766	3.161032	16.82835	4.247696	23.52466	3.775072
437	209	4.135163	1.629411	8.082916	2.645419	16.59295	4.090419	23.27365	3.873926
438	208	3.703292	1.537237	8.082639	2.559838	16.61538	3.932803	23.03497	3.784351
439	207	4.417326	1.458241	7.977119	2.795361	16.36312	3.811016	22.64707	3.479995
440	206	3.916754	1.277026	7.819076	2.812376	16.28709	3.935565	22.77706	3.783673
441	205	3.992991	1.392871	8.368744	2.812724	16.99116	3.75556	23.40598	3.328916
442	204	3.953265	1.644774	8.876973	2.552682	16.15538	3.766908	22.77985	3.788738
443	203	4.407163	1.525641	8.31963	2.659569	16.41692	3.963757	23.00693	3.850176
444	202	3.964785	1.522796	8.249122	3.018403	16.62362	3.715143	22.25099	3.894977
445	201	4.275099	1.481206	8.77632	2.675731	17.06521	3.545973	23.05074	3.809027
446	200	4.28661	1.676745	8.821354	3.09835	16.9971	3.995977	23.58889	4.152794
447	199	4.319309	1.506751	7.943063	2.941297	17.19093	3.791363	24.20332	4.000056
448	198	4.235789	1.666757	8.20954	2.804767	17.38891	4.354394	23.89069	4.09656
449	197	4.000659	1.779525	8.710119	3.043805	17.13533	3.942172	22.4382	4.065816
450	196	4.312602	1.603419	9.133716	2.718705	16.41906	3.824416	22.28673	3.492645
451	195	4.029041	1.45328	8.776035	2.551925	15.68534	4.005118	22.78998	3.585797
452	194	4.260644	1.251306	8.419191	2.717673	16.14197	4.156032	22.93376	3.960764
453	193	4.286358	1.315411	8.285541	2.355738	15.22205	4.390189	22.24068	3.784537
454	192	4.223301	1.482112	8.410444	2.39224	15.6446	4.094006	23.09334	3.824935
455	191	4.273193	1.520739	8.119695	2.801219	15.97054	3.61731	23.14465	3.821703
456	190	4.419578	1.451377	8.169705	2.697464	15.37695	3.688167	23.2176	3.900835
457	189	3.92144	1.525952	7.807369	2.639608	16.16989	3.635555	22.57009	4.182722
458	188	4.171096	1.439505	7.893022	2.636935	15.22349	3.70514	22.47016	3.554921
459	187	3.882499	1.599813	8.330019	3.175248	16.07569	3.9122	22.24903	4.057843
460	186	3.966182	1.39974	8.317902	3.091164	16.08324	3.993375	22.36585	3.60259
461	185	4.182779	1.56907	8.062231	2.490299	15.92035	3.532675	22.19704	3.856373
462	184	4.473775	1.702006	8.376324	2.729642	15.37506	3.749513	23.2682	3.954091
463	183	3.998259	1.381472	8.274545	2.580703	15.65321	3.475954	22.71894	3.649218
464	182	3.694321	1.310576	8.627945	2.472624	15.39516	3.873685	23.40259	3.307543
465	181	3.889418	1.640351	8.987352	2.746913	16.46165	3.719889	22.8876	3.598545
466	180	4.25894	1.611059	8.43677	2.817496	15.74826	3.934286	22.4077	3.674978
467	179	4.186656	1.265987	8.621249	2.705121	15.80394	4.20415	22.02341	3.889869
468	178	4.021248	1.321209	8.327246	2.532811	15.19999	3.67897	22.04625	3.886302
469	177	4.136251	1.779147	8.062933	2.747896	14.92712	3.609146	22.1356	3.662464
470	176	4.004775	1.329676	8.617178	2.941297	14.99082	3.184105	23.10513	3.142833
471	175	4.164233	1.404956	9.152326	2.989745	14.69243	3.919029	22.43239	3.894559
472	174	4.354938	1.234034	9.069268	2.826507	14.44895	3.886146	22.42099	3.299989
473	173	3.93429	1.547835	8.383914	2.508075	15.24464	3.579382	22.37327	3.411818
474	172	4.233823	1.30956	8.41569	2.960767	14.64987	3.725519	21.74399	3.652652
475	171	3.491875	1.304033	9.132784	2.545563	14.97323	3.282196	21.84234	3.646778
476	170	4.426606	1.408472	8.245262	2.81927	14.54441	3.576886	21.99085	3.717005
477	169	4.401728	1.489286	8.300714	3.010174	15.52619	3.639179	21.35368	3.457205
478	168	4.094271	1.49874	8.69466	2.74133	15.04657	3.414859	22.3027	3.816541
479	167	4.037091	1.582164	8.138161	2.701455	15.30993	3.847915	22.26753	3.817774
480	166	4.160157	1.564901	9.172802	2.533988	15.12569	3.838074	21.64504	4.070126
481	165	4.293644	1.638226	8.628169	2.903898	15.07853	3.73652	22.64513	3.787976
482	164	4.212432	1.152972	8.451262	2.749988	15.06175	3.682255	22.44989	3.790466
483	163	4.046606	1.498249	8.053178	2.862215	15.18322	3.91449	22.32654	3.748958
484	162	4.545528	1.350083	8.528481	2.936881	15.27149	4.034309	23.27445	3.740766
485	161	4.383647	1.143221	8.898777	2.751035	14.61852	4.029768	23.88778	3.82184
486	160	4.295432	1.566373	9.128284	2.773446	14.56126	3.082372	22.31851	3.38665
487	159	3.903523	1.072615	8.992698	2.499355	13.91105	3.245558	22.44941	3.553799
488	158	3.700677	1.403521	8.381767	2.703055	13.13654	3.524472	20.52895	3.569447
489	157	3.609926	1.319916	8.399787	2.566678	13.09868	3.333639	20.01269	3.41844
490	156	3.584083	1.14416	8.42159	2.428253	13.12397	3.277225	19.82413	3.223073
491	155	3.839716	1.327549	7.69421	2.279534	12.8035	2.856636	19.3561	3.156521
492	154	3.493418	1.197717	7.650562	2.176077	12.83343	3.036042	19.10921	3.061268
493	153	3.254908	1.094785	7.628684	2.257503	12.23128	3.068953	19.53096	3.156295
494	152	3.671521	1.345065	7.465216	2.442325	11.77716	2.923587	18.73005	3.121981
495	151	3.614865	0.928923	7.58462	2.236572	12.18123	3.375069	18.11027	3.031067
496	150	3.376958	1.131349	7.687169	2.408215	12.36057	2.65889	18.53381	2.716627
497	149	3.245636	1.048517	7.577093	2.257246	12.36972	2.94708	18.43126	2.791706
498	148	3.372591	1.05533	7.459936	2.364038	12.68769	3.257276	18.01716	3.357171
499	147	3.382873	1.478529	7.19657	2.372014	12.83838	2.637016	19.63802	3.232549
500	146	3.842535	1.038568	7.366006	2.465422	13.47604	3.078028	19.5825	3.795259
501	145	3.667953	1.474656	7.209218	2.763862	14.07833	3.583202	19.75855	3.887637
502	144	3.869754	1.152217	7.736173	2.471894	14.58723	3.474524	20.62626	4.005753
503	143	3.783366	1.58733	8.097386	2.607463	15.01628	3.581302	21.33693	4.120375
504	142	4.180137	1.377472	8.658786	2.950621	15.63191	3.575484	22.47512	4.086367
505	141	4.759389	1.241954	9.801737	3.071545	15.87709	3.265773	23.98738	3.534881
506	140	4.899109	1.528194	10.75932	2.946445	15.61965	3.419662	24.20779	3.214151
507	139	4.761491	1.550225	10.78763	2.855198	13.2625	3.218791	23.10785	3.362037
508	138	4.074846	1.20958	9.369583	2.681622	13.47222	3.197919	20.35437	3.110221
509	137	3.589862	1.359949	8.905953	2.586495	11.72278	2.84984	18.76169	3.03436
510	136	3.314683	1.101753	7.664995	2.584634	11.16219	2.902303	17.38666	2.638041
511	135	3.121444	0.964191	7.307546	2.401806	10.95455	2.382181	16.19363	2.722
512	134	3.228946	0.875577	7.031317	2.290063	9.910894	2.498271	15.5189	2.452787
513	133	2.898229	0.619484	6.387322	1.763986	9.564419	2.444261	14.66698	2.340559
514	132	2.636623	1.001117	6.029182	2.054126	8.374236	2.477959	14.3086	2.121062
515	131	2.592192	0.630724	5.760093	1.655385	8.264932	2.070944	13.28218	1.743193
516	130	2.892461	0.839709	5.747486	1.80081	8.010946	2.15889	13.19988	2.037397
517	129	2.206577	0.598467	5.019444	1.61102	7.757945	1.823063	12.45341	1.857619
518	128	2.597888	0.736846	5.27392	1.797003	7.234684	1.873024	11.78906	1.812701
519	127	2.634672	0.563042	5.181672	1.368463	7.137302	1.888704	12.17906	1.641608
520	126	2.242582	0.645347	5.178856	1.462362	7.122938	2.090736	11.99501	1.898515
521	125	2.180624	0.644213	5.08222	1.594826	6.582396	1.869912	12.08862	1.516404
522	124	2.396113	0.588353	4.902801	1.694913	7.946516	1.937466	12.42415	1.683849
523	123	2.426509	0.702938	5.387418	1.736766	8.093674	2.159679	13.21372	1.676889
524	122	2.856479	0.796497	5.619859	1.581186	8.032481	1.908863	13.62056	1.851942
525	121	2.365754	0.760774	5.56516	1.438888	7.511861	1.999097	12.97408	1.78487
526	120	2.462533	0.564124	5.298657	1.285654	6.98356	1.851426	12.41583	1.718221
527	119	2.398758	0.60893	4.760032	1.428565	6.756207	1.998892	11.16601	1.418339
528	118	2.082062	0.574979	4.672902	1.406124	6.573645	1.58583	10.49723	1.357548
529	117	2.158988	0.57383	4.034268	1.426092	6.654795	1.724285	11.03531	1.582623
530	116	2.158456	0.782802	4.327334	1.572286	6.495295	1.526971	10.86987	1.470422
531	115	2.017875	0.484347	4.329304	1.551221	6.021198	1.704741	10.47929	1.303499
532	114	2.221714	0.70337	4.174169	1.37142	6.083372	1.808046	10.67387	1.395543
533	113	2.436745	0.565976	4.167051	1.571023	6.601718	1.691673	10.41914	1.37894
534	112	2.130509	0.708215	4.481077	1.178915	6.027672	1.767489	10.4294	1.490191
535	111	2.22774	0.590701	4.820784	1.340414	6.266618	1.45702	9.3317	1.453034
536	110	1.904886	0.565535	4.554726	1.415224	5.891252	1.669931	10.71125	1.430392
537	109	2.336201	0.566418	4.68556	1.32092	6.407591	1.5607	10.22338	1.448051
538	108	1.967399	0.54101	4.567259	1.359207	6.096896	1.708776	9.861004	1.30825
539	107	2.182267	0.485421	4.479677	1.443033	6.330146	1.973633	9.843028	1.307362
540	106	2.135396	0.6109	4.828853	1.584868	6.192212	1.809911	9.957658	1.333191
541	105	2.17831	0.601467	4.411131	1.500819	6.062991	1.404228	10.37915	1.213341
542	104	2.129838	0.681146	4.640992	1.536082	6.687312	1.52965	10.16854	1.506395
543	103	2.167396	0.53233	4.338343	1.683334	7.300899	1.749913	10.58005	2.025
544	102	1.756805	0.68367	4.618775	1.627438	7.382619	1.819965	11.55575	1.825598
545	101	2.225443	0.624986	4.593597	1.540464	7.743984	1.666851	11.138	2.296193
546	100	2.158322	0.599594	4.639504	1.273319	7.50588	1.686815	10.56611	2.06357
547	99	2.01801	0.543857	4.328219	1.45262	6.283167	1.445502	10.51148	1.477081
548	98	2.330953	0.49704	4.356509	1.469001	6.148101	1.328399	10.67633	1.364127
549	97	2.324493	0.646459	4.082067	1.504001	5.753576	1.449047	9.601265	1.354071
550	96	1.952011	0.531303	4.495056	1.611635	6.129396	1.549545	9.616782	1.461417
551	95	2.006113	0.589005	4.285173	1.480693	6.136518	1.63806	10.05624	1.410622
552	94	2.090068	0.586023	4.050929	1.329973	5.932554	1.657618	9.539506	1.235117
553	93	1.801857	0.576746	4.719819	1.132672	5.737655	1.393105	9.299131	1.279157
554	92	2.022754	0.494441	4.164945	1.324335	5.620545	1.423743	8.966446	0.89812
555	91	1.993184	0.64395	3.928052	1.270822	5.471497	1.627975	8.439581	1.007138
556	90	1.797809	0.380045	4.072034	1.408688	5.394133	1.336346	9.351661	1.182413
557	89	1.959802	0.598638	4.211449	1.028066	5.075672	1.443164	8.709719	1.220253
558	88	1.858126	0.657167	4.125034	1.545644	5.186489	1.368928	8.594881	1.120007
559	87	1.90877	0.461775	4.434981	1.433902	5.093653	1.305588	8.061454	1.050091
560	86	1.698717	0.485421	4.009014	1.404934	4.908159	1.228979	8.415142	1.131517
561	85	1.503154	0.323455	3.788815	1.096857	5.049705	1.06126	7.858718	0.860037
562	84	1.742571	0.381215	3.852282	1.370549	5.354321	1.149469	8.119276	1.093421
563	83	1.44775	0.588329	4.167304	1.441934	4.531601	1.314716	7.482712	0.940475
564	82	1.544649	0.416092	3.862737	1.479312	5.174204	1.225398	8.1871	1.032304
565	81	1.690093	0.49651	3.947508	1.419362	4.729124	1.303125	7.747474	1.035021
566	80	1.902446	0.462401	4.19323	1.32092	5.168649	1.486578	8.121377	1.022662
567	79	1.985101	0.438938	4.607425	1.523223	5.683203	1.618836	9.142601	1.092557
568	78	2.326034	0.450046	5.168459	1.472758	6.494016	1.78963	10.44377	1.287635
569	77	2.469188	0.704207	5.323681	1.203139	7.529014	1.789814	12.35584	1.706648
570	76	2.790828	0.672941	5.893598	1.480567	8.28489	2.141888	14.40409	1.717358
571	75	3.336664	0.821504	6.5818	1.455206	8.814747	2.154516	15.10869	1.729369
572	74	3.004187	0.682939	5.893598	1.665887	8.604881	2.339181	13.47571	1.651979
573	73	2.450204	0.601195	4.845734	1.408569	7.053177	1.789691	11.16129	1.413027
574	72	1.68957	0.520543	3.710725	1.139284	4.862983	1.473945	8.058265	1.180083
575	71	1.487253	0.24323	3.342376	1.032759	4.020942	1.230417	6.265776	0.847877
576	70	1.146639	0.382219	2.929468	1.236066	4.087601	1.074037	5.781832	0.908342
577	69	1.194219	0.288953	2.825654	1.212168	3.80471	1.035653	5.423678	0.714404
578	68	1.303815	0.437896	2.743238	1.157057	3.555549	0.957102	5.252325	0.727012
579	67	1.294596	0.334102	3.062189	1.229722	3.670332	0.880819	5.306355	0.667818
580	66	0.9199342	0.288823	2.643895	0.978587	3.415846	0.900859	4.707104	0.710129
581	65	1.192638	0.345849	2.825536	1.337117	3.578188	0.793869	4.828863	0.656684
582	64	1.0425	0.230255	3.074434	1.316198	3.247186	0.870033	4.824718	0.61747
583	63	0.9685908	0.28855	3.265344	1.396454	3.45729	0.973895	4.790935	0.717147
584	62	0.8936502	0.346346	2.975224	1.243983	3.354604	0.984921	4.533681	0.74174
585	61	1.192796	0.277657	2.95401	1.32383	3.667576	0.887417	4.648139	0.607063
586	60	1.093638	0.300993	3.381222	1.23155	3.625243	0.878403	4.73967	0.786215
587	59	0.9854868	0.278413	3.064616	1.160746	3.733235	1.025868	4.329622	0.746538
588	58	0.9485677	0.173736	2.918965	1.244353	3.199413	0.839533	4.65732	0.717198
589	57	0.9726242	0.359083	2.906778	1.195629	3.276808	0.92654	5.205498	0.769971
590	56	1.065054	0.267021	2.527338	1.312738	3.088587	0.937284	4.772611	0.82873
591	55	0.8966551	0.220056	2.676689	1.232283	3.239257	0.839073	4.563976	0.655978
592	54	1.094895	0.323614	2.762083	1.137297	3.046407	0.878343	4.480974	0.676963
593	53	0.9606655	0.34813	2.386577	0.894897	3.18419	0.918148	4.911123	0.777974
594	52	1.042914	0.30103	2.91116	1.158382	2.888432	0.70458	4.577395	0.567224
595	51	0.8812772	0.196529	2.365631	1.121034	2.777683	0.957069	4.857307	0.791201
596	50	0.7102605	0.196174	2.184617	0.714827	2.907193	0.830708	4.339725	0.538562
597	49	1.083554	0.299503	2.08987	1.095187	2.890227	0.87252	4.52005	0.618916
598	48	0.8710543	0.311596	1.933368	0.798528	2.862708	0.84396	4.567041	0.771442
599	47	0.9984923	0.265108	1.943644	0.822051	2.999505	0.84161	4.463283	0.667434
600	46	0.9679929	0.230925	2.082211	0.679402	2.77832	0.708003	4.449213	0.649168
601	45	0.8221521	0.266581	1.856912	0.846667	2.78241	0.727025	4.371346	0.698927
602	44	0.769159	0.28931	2.023054	0.667709	2.480118	0.717257	4.108034	0.585901
603	43	1.081926	0.231457	1.994612	0.71613	2.536804	0.649319	4.197113	0.657628
604	42	0.9437968	0.347072	1.669816	0.680555	2.381097	0.746233	4.285713	0.548071
605	41	0.9243431	0.242621	1.95876	0.587781	2.64356	0.522774	4.372989	0.701527
606	40	0.872984	0.290037	2.315071	0.778647	2.740805	0.72665	4.358928	0.527715
607	39	0.7601706	0.197453	2.490097	0.756105	2.714099	0.687303	4.262358	0.576772
608	38	0.9732279	0.291045	3.947064	1.716311	2.882874	0.726325	4.250628	0.729184
609	37	1.046375	0.278918	4.920029	2.453958	3.392816	1.019731	4.375622	0.599337
610	36	0.8460175	0.162354	5.742	2.36463	3.700718	0.869675	4.517977	0.69181
611	35	1.144612	0.232144	6.219341	3.183077	3.968196	1.198836	4.329159	0.731473
612	34	0.9081443	0.428989	4.738162	2.253238	3.858716	0.900364	4.474642	0.690512
613	33	0.9704747	0.19725	2.320525	1.518126	2.582002	0.628551	4.107307	0.629176
614	32	0.8095877	0.173893	1.519195	1.013444	2.690984	0.766011	4.076608	0.57671
615	31	0.8721338	0.299454	1.327047	0.86067	2.791701	0.659464	4.130266	0.496125
616	30	0.9099541	0.253945	1.416153	0.4192	2.642614	0.591578	4.182288	0.628768
617	29	0.9127836	0.184491	1.225597	0.273461	2.594664	0.580381	4.034129	0.558321
618	28	0.8203731	0.231886	1.383227	0.29892	2.404828	0.58044	4.061073	0.586786
619	27	0.8099103	0.24305	1.509345	0.214976	2.178485	0.48395	4.184504	0.574583
620	26	0.8130777	0.230519	1.391281	0.333121	2.470288	0.630067	3.923586	0.667242
621	25	0.7088171	0.345991	1.399086	0.310323	2.390835	0.484917	3.957572	0.567122
622	24	0.9714623	0.231267	1.466199	0.286003	2.634308	0.747597	4.120896	0.506888
623	23	0.7612829	0.207782	1.373449	0.202406	2.324645	0.495264	4.057441	0.546278
624	22	0.7219222	0.24345	1.280233	0.226132	2.349386	0.572281	3.956336	0.537166
625	21	0.7358986	0.219523	1.237175	0.166906	2.077954	0.746978	3.619488	0.405087
626	20	0.7362575	0.18493	1.467238	0.227121	1.79424	0.590785	3.757242	0.586701
627	19	0.8628065	0.230746	1.03648	0.274983	2.18412	0.552634	3.750348	0.476492
628	18	0.7964629	0.172854	0.978326	0.203136	2.26758	0.551874	3.43868	0.507729
629	17	0.8221521	0.149807	1.119396	0.215543	1.913795	0.533152	3.371554	0.609935
630	16	0.8082644	0.207978	1.003034	0.310811	1.964294	0.473587	3.693037	0.46728
631	15	0.5601369	0.138572	1.155422	0.334109	1.693803	0.58068	3.299894	0.447157
632	14	0.6210285	0.162387	1.061992	0.143793	1.724589	0.436772	2.887535	0.294589
633	13	0.6336169	0.150416	1.171038	0.214976	1.769414	0.474581	3.029397	0.314679
634	12	0.4856452	0.127438	0.977221	0.155339	1.426377	0.338835	2.79555	0.264783
635	11	0.6453856	0.161277	0.882994	0.131726	1.389251	0.378079	2.467595	0.314565
636	10	0.5100085	0.127281	0.802865	0.155538	1.471861	0.368944	2.584975	0.304385
637	9	0.5103243	0.149966	0.919891	0.203568	1.668095	0.368766	2.393884	0.546573
638	8	0.5847474	0.138436	0.844319	0.287427	1.541273	0.418167	2.492248	0.262485
639	7	0.7098205	0.069218	0.996766	0.251328	1.799324	0.349249	2.722961	0.414961
640	6	0.6620883	0.185112	1.341141	0.203716	1.809745	0.523079	3.095867	0.475772
641	5	0.9749999	0.289191	1.702848	0.358689	2.874262	0.68109	4.581203	0.696645
642	4	1.71973	0.670371	2.79642	0.441483	4.569188	1.236632	7.237672	1.152633
643	3	2.523491	0.635645	4.679297	0.441745	7.157082	1.696157	12.2203	1.608313
644	2	2.540523	0.53139	4.320423	0.514755	6.732223	1.697971	12.88424	1.353148
645	1	1.270205	0.1969	2.870273	0.287415	3.458376	0.952834	6.993722	0.671705
646	0	0.4371893	0.138983	0.742771	0.16768	1.325628	0.271666	2.497093	0.233438
647	−1	0.1617172	0.023172	0.18849	0.060042	0.322096	0.116224	0.807004	0.040542
648	−2	0.049834	0.023174	0.070693	0.024004	0.046166	0.009713	0.139586	0
649	−3	0.0249446	0	0.070912	0.011961	0.027672	0.009698	0.099694	0.020201
650	−4	0	0	0.035261	0.011988	0.045855	0.009662	0.05959	0.040411

XPS measurements were conducted using photon energy of 650eV throughout the whole experiments. The gold (Au) spectra was determined before canning each sample in order to acquire the reference point and equipment standard for energy shifting during analysis. Sample spectra were analyzed using excel with the macro code developed for XPS specially in the SLRI facility. Core scans of W and C were determined in order to comprehend phase and structural changes within the film.

**Fig. 1:**
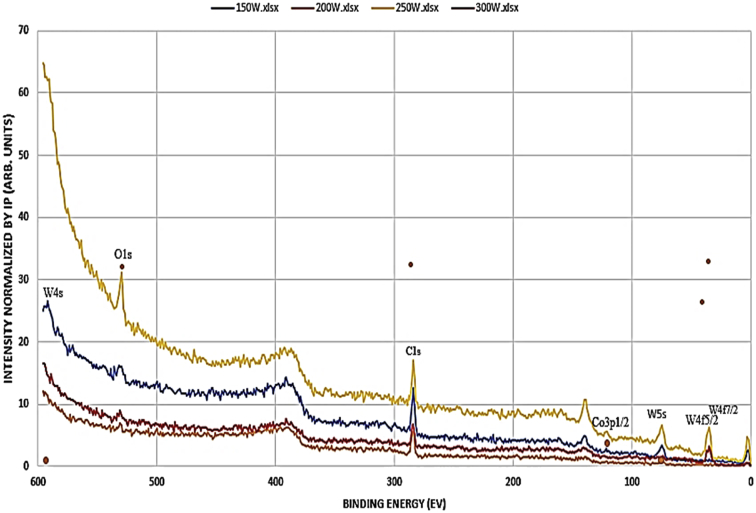
XPS spectra for WC-Co thin film deposited at various rf power settings.

**Fig. 2:**
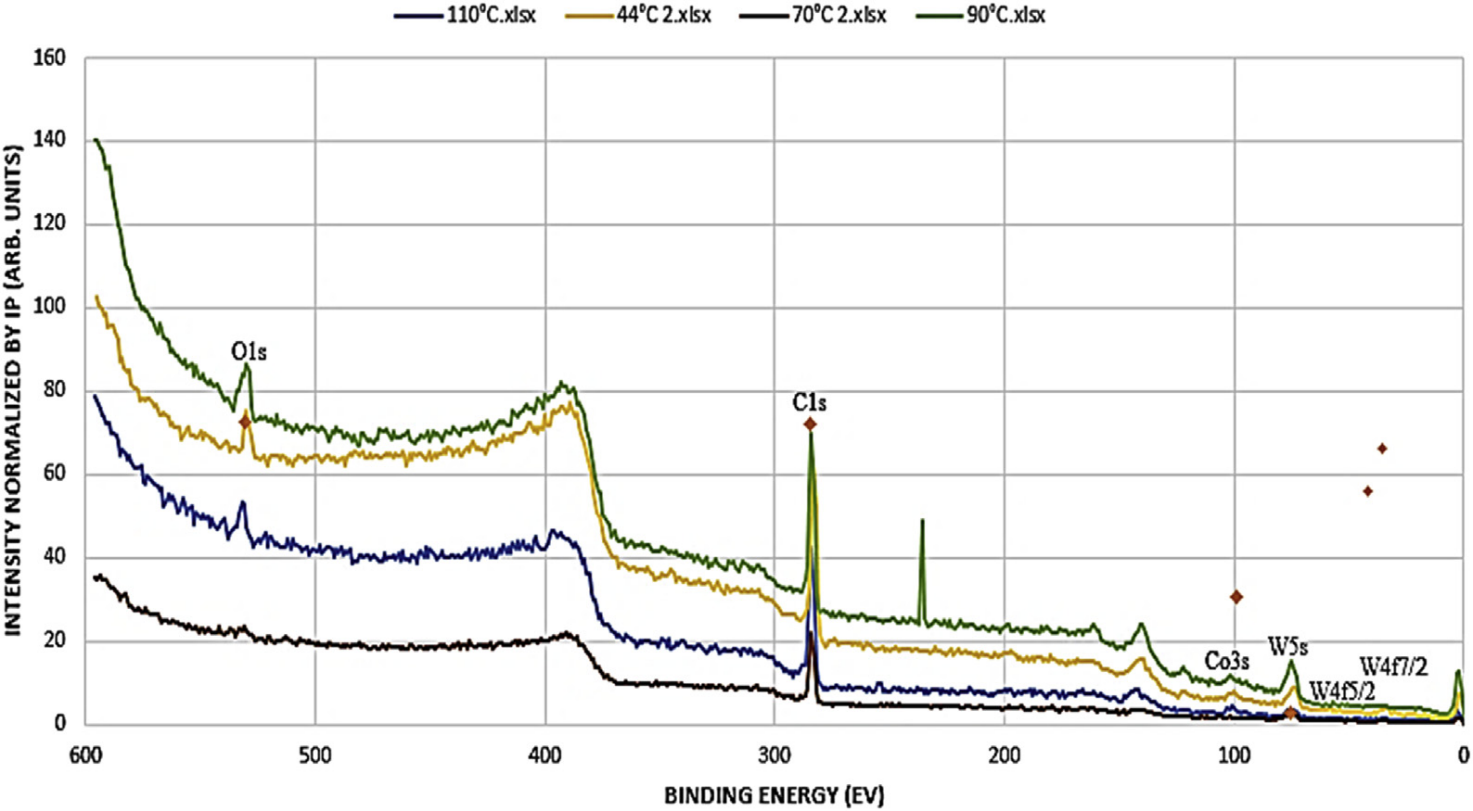
XPS spectra of WC-Co thin film deposited at varying temperature.

**Fig. 3:**
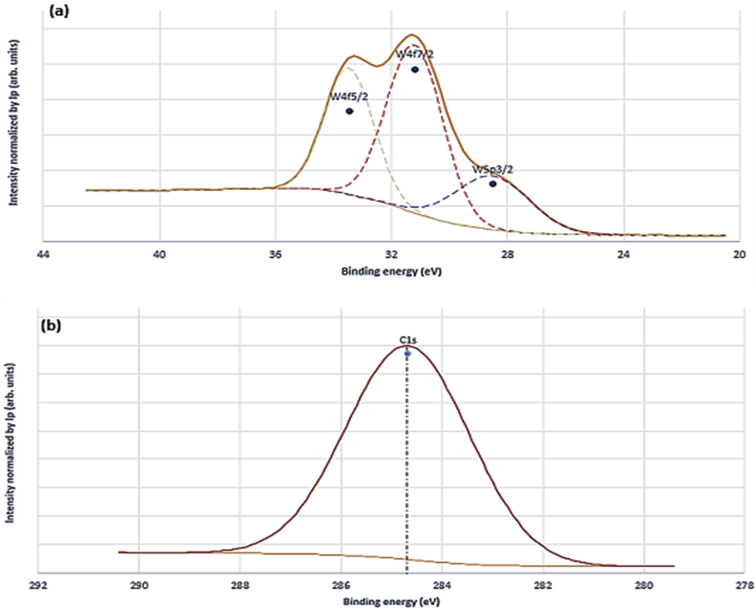
High resolution core spectra of WC samples at (a) W4f region and (b) C1s region.

**Fig. 4:**
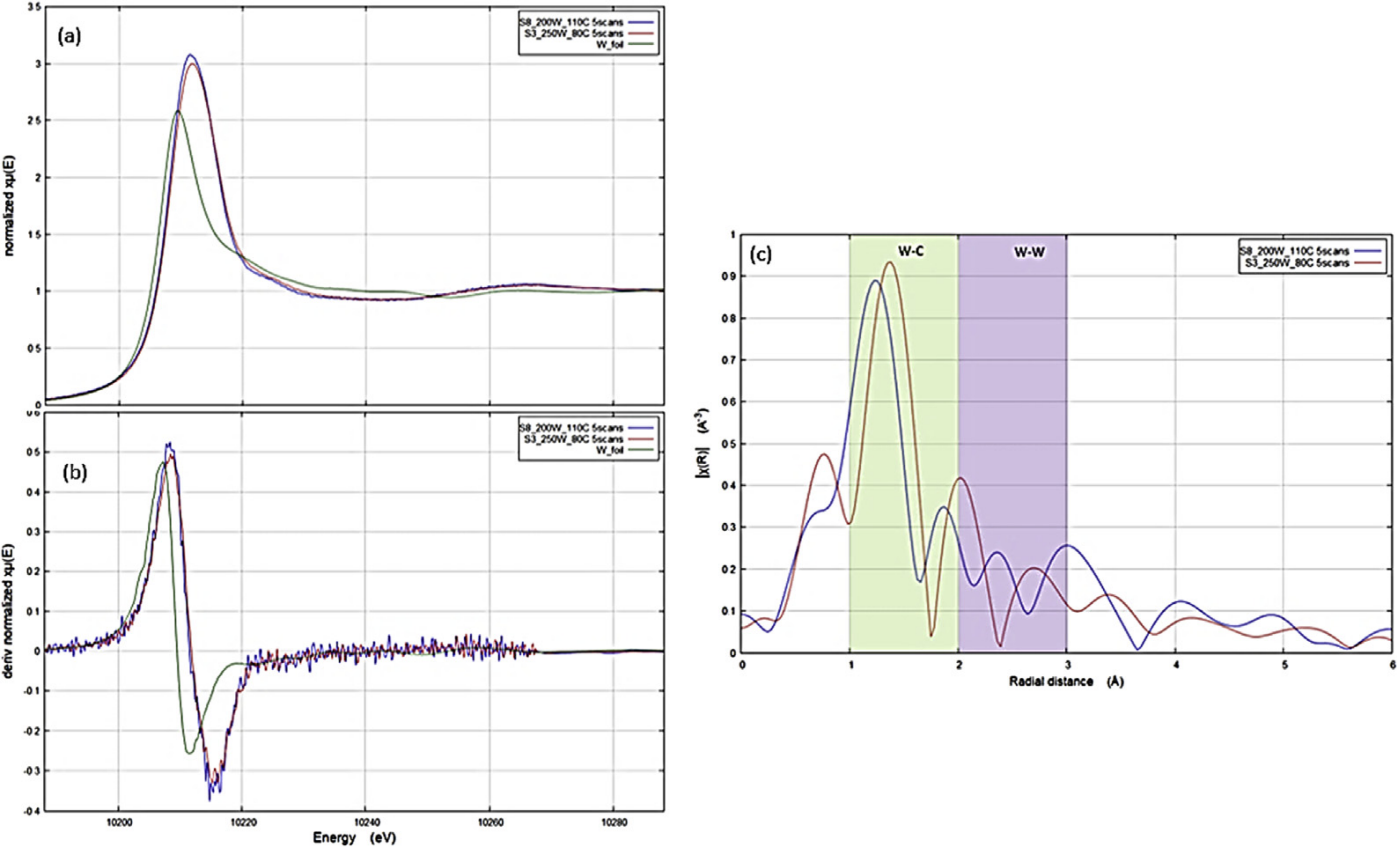
WC L edge GI-XAS spectra for W foil and WC thin films. (a) Normalized GI-XAS spectra, (b) 1st derivative of normalized spectra showing absorption edge and (c) Fourier transform magnitudes.

The x-ray absorption of the film was conducted using beamline 1.1XAS of the SLRI employing glazing incidence mode at an angle of 0.4° due to the small thickness of the film. One sample at a time was loaded and fixed to the goniometer to allow for precision alignment of the sample, source and detector. The detector was positioned 90° to the sample such that it does not take readings from the radiating source but rather detect the scattered irradiated electrons from the sample. It was ensured that the sample was perfectly smooth and level in order to acquire accurate readings since the gracing incidence technique is very sensitive to surface profiles [1]. The W foil was used as a standard material for the test. The obtained spectra were analyzed using the Athena software, see Fig. 1, Fig. 2, Fig. 3, Fig. 4.

**Fig. 5:**
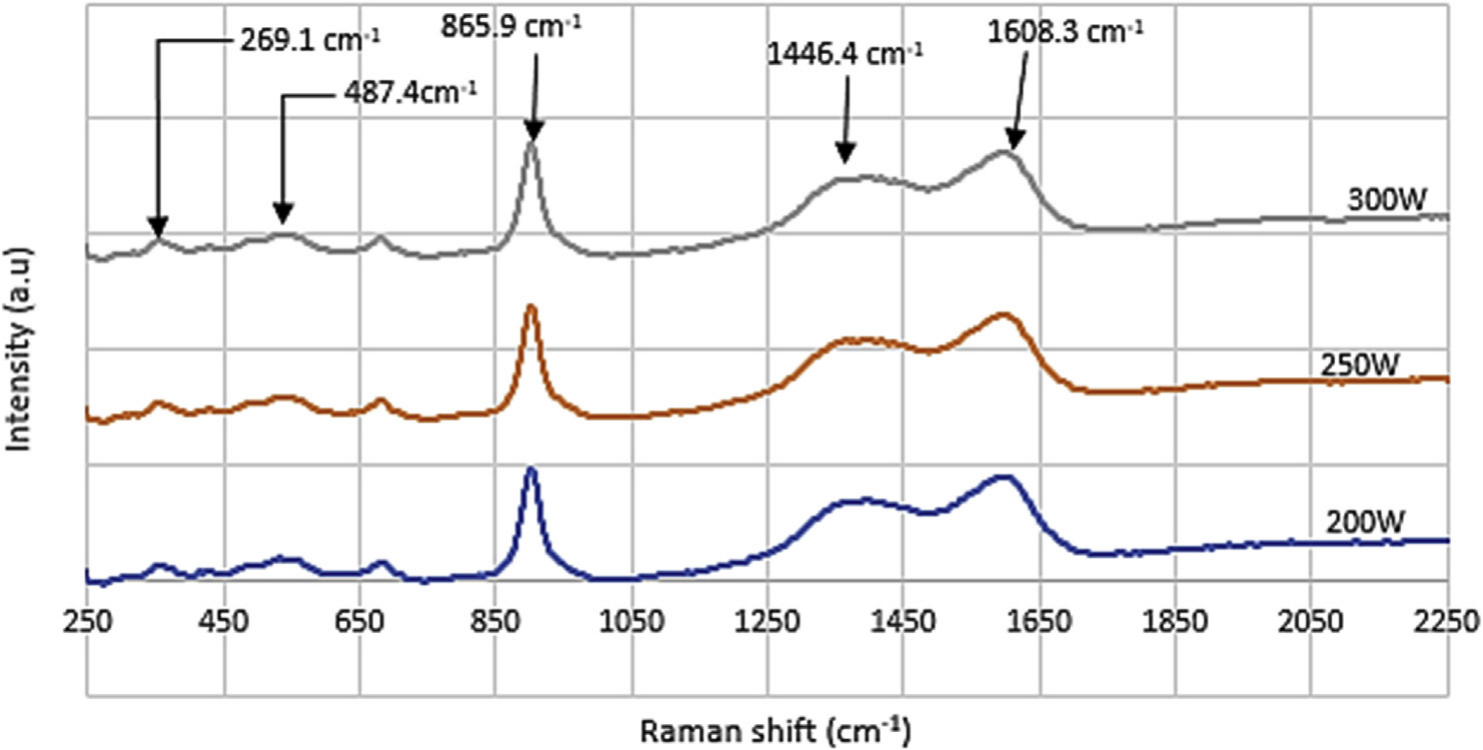
Raman spectra of WC films deposited at various RF powers.

**Fig. 6:**
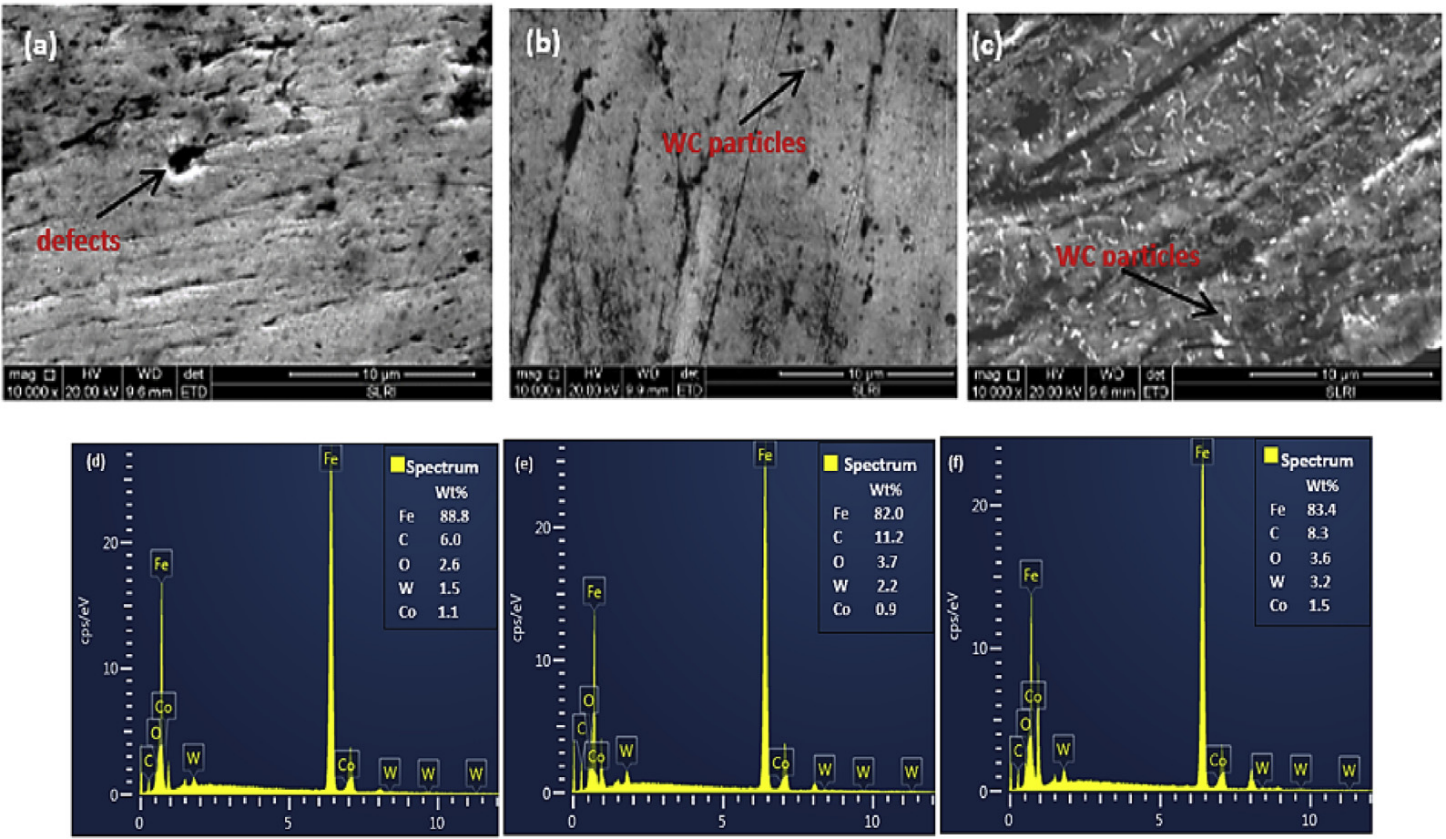
Surface SEM images surface for deposition temperature (a) 70 °C (b) 90 °C (c) 110 °C and their respective EDS spectra (d)(e) and (f).

**Fig. 7:**
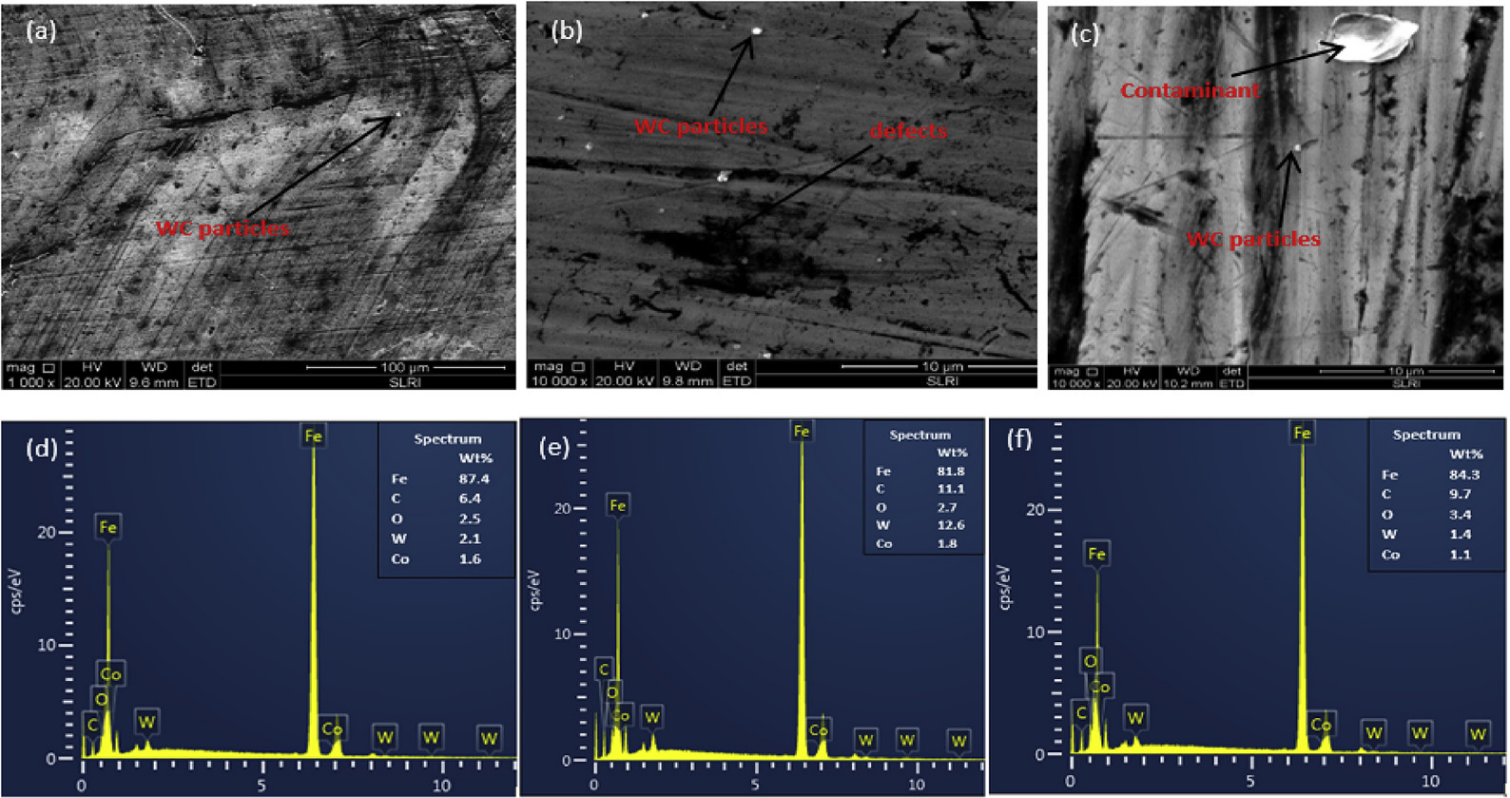
Surface SEM images surface for deposition at RF powers (a) 200W (b) 250W (c) 300W and their respective EDS spectra (d)(e) and (f).

The film microstructure, surface topography and phase identification were validate using SEM (see Fig. 6, Fig. 7), profilometry and Raman spectroscopy (see Fig 5) respectively. Surface roughness properties of the thin film are presented in Table 4. Detailed complimentary procedures and analysis are explained elsewhere [2], [3], [4], [5].

**Table 4:** WC thin film surface roughness properties.

Sample	Roughness Properties						
	Sa(nm)	Sku	Sp(nm)	Sq (nm)	Ssk	Sv(nm)	Sp(nm)
150W	433.7 ± 29.5	8 ± 2.9	4479.5 ± 171.8	583.6 ± 32.4	−1.2 ± 0.4	−6012.67 ± 791	10492.2 ± 784.3
200W	204.1 ± 54.6	62.5 ± 25.8	4349.8 ± 109	313.6 ± 75.9	−2.23 ± 0.5	−7053.9 ± 1902.3	11404.7 ± 1943
250W	484.2 ± 27.5	9.2 ± 2.3	4873.4 ± 345.7	658.5 ± 41.8	−1.4 ± 0.3	−6397 ± 899	11270.4 ± 1192
300W	379.6 ± 15.8	5.4 ± 0.1	4630.8 ± 294.8	497.6 ± 18	−0.52 ± 0.1	−4848.9 ± 496.6	9479.7 ± 553
44ׄ°C	136.6 ± 24	89.5 ± 40.5	3808.2 ± 549.6	201.1 ± 23.7	1.2 ± 1.9	−8583.5 ± 796.3	12391.7 ± 289.4
70 °C	263.2 ± 31.6	22.6 ± 4.4	4456.8 ± 174.2	429.4 ± 66.8	−2.5 ± 0.9	−7487.6 ± 1317.9	11944 ± 1321.1
90 °C	252.2 ± 21.3	5.8 ± 0.1	4128 ± 369.6	337.8 ± 26.1	−0.26 ± 0.1	−4402.9 ± 138.2	8530.9 ± 506
110 °C	310.4 ± 2	5.49 ± 0.3	4351.5 ± 133	402.23 ± 2	−0.124 ± 0.09	−5518.66 ± 1232.6	9870.19 ± 1355.1
